# Induction of secondary axis in hydra revisited: New insights into pattern formation

**Published:** 2012

**Authors:** Vishal Kadu, Saroj S. Ghaskadbi, Surendra Ghaskadbi

**Affiliations:** 1 *Division of Animal Sciences, Agharkar Research Institute, Pune, India *; 2 *Department of Zoology, University of Pune, Pune, India*; 3 *Division of Animal Sciences, Agharkar Research Institute, Pune, India*

**Keywords:** Hydra, interspecific grafting, morphogen gradients, organizer, induction of secondary axis

## Abstract

In 1909, several years before the famous `Organizer’ experiments of Spemann and Mangold, Ethel Browne demonstrated induction of a secondary axis in hydra by grafting a hypostome. Based on this and subsequent work, in the late sixties, Lewis Wolpert proposed the theory of morphogen gradients and positional information. We have studied secondary axis induction by hypostome and foot tissue using three species of hydra as well as transgenic, GFP-expressing lines of hydra. We have found that pieces of hypostome and complete foot of a donor hydra can induce a secondary axis all along (in upper, middle or lower parts of) the body column of a host hydra, both within and across species with comparable rates. Thus, contrary to the available literature, our results show that the host hypostome does not completely inhibit the induction of a secondary axis. The length of the induced axis though is determined by the position of the graft. By using GFP-expressing lines of hydra we have demonstrated that host ectodermal and endodermal cells actively contribute to the secondary axis. On comparison, the hypostome was found to be a stronger and dominant Organizer than the foot. Foot grafting experiments show a transient increase in the host length as well as the distance between the two Organizers. The length becomes normal once the grafted foot reaches the budding zone. Our work brings out several new aspects of the role of positional cues in pattern formation in hydra that can be now be explored at cellular and molecular levels.

Hydra, a common freshwater Cnidarian (family Hydridae, class Hydrozoa), was first described by the Swiss naturalist Abraham Trembley in 1744 and classified by Carl Linnaeus in 1758. The body of a hydra, with a single oral-aboral axis, consists of a hypostome surrounded by tentacles, body column, bud(s) and foot (sometimes with peduncle) along it. This axis is called the primary axis and any deviation in this axis due to formation of a new head or foot structure results in the formation of a secondary axis. Using the lateral grafting technique, it has shown that the hypostome, peduncle and basal disc (or foot) could induce a secondary axis when grafted onto the host of the same species (homoplastic transplantation) (1-3). The head organizer located in the hypostome and two morphogenetic gradients, head activator (HA) and head inhibitor (HI) gradients, that run the length of the oral-aboral axis play essential roles in axis formation of hydra (4,5). A second organizer is known to reside in the foot. Lateral transplants of the two different organizer tissues, when transplanted together onto the middle part of host, antagonized each other’s inductive ability (6). A piece of hypostome could induce secondary axis when transplanted within the species (7). By using a labeled transplant and an unlabeled host, it has been shown that cells from the host migrate into the secondary axis and support its growth (7). A full understanding of the underlying mechanisms of axis induction in hydra, however, remains elusive.

We have reexamined the various aspects of secondary axis induction in hydra with a view to further exploring hydra as a model system to study pattern formation. In the present study, we have attempted to do the following: 1. Compare the inductive abilities of hypostome and foot, 2. Study the interaction between the hypostome and foot Organizers, 3. Study the influence of host hypostome on induction of secondary axis, 4. Compare secondary axis induction within and between species of hydra and 5. Study the contribution of host cells to secondary axis formation. 

We have found that when either a piece of hypostome or a foot is grafted on different parts of the host body column within or across species, a secondary axis is induced, the length of which varies depending upon the position of grafting. The inducing capacity of hypostomal tissue is much greater than foot tissue since only a small piece of hypostome induces a second axis while the whole foot is required for induction to occur. The hypostomal and foot organizers seem to actively interact with each other as evident from the active away migration of foot grafted near host hypostome. This migration continues till the new axis reaches the budding zone or just below it, presumably when normal positional information gradients are reestablished. We have also found, contrary to earlier reports, that a secondary axis is induced even in the presence of the host hypostome though the length of the induced axis is drastically reduced. Finally, by using a combination of non-transgenic grafts and transgenic hosts, we confirm that the host cells (both ectodermal and endodermal) contribute to the secondary axis.

## Materials and methods

**Hydra culturing and maintenance**

Polyps were cultured at 18°C. The animals were fed with freshly hatched Artemia nauplii and the medium was changed daily. Animals starved for 24 hrs were used in all experiments. Transplantation experiments were carried out by using Hydra vulgaris Ind-Pune (8), H. magnipapillata, H. vulgaris (AEP) and H. vulgaris (AEP) expressing GFP either in its ectodermal or endodermal cells (ecto and endo lines) respectively; a kind gift from Prof. Thomas Bosch, University of Kiel, Germany (9).

**Grafting procedure**

**Fig 1:**
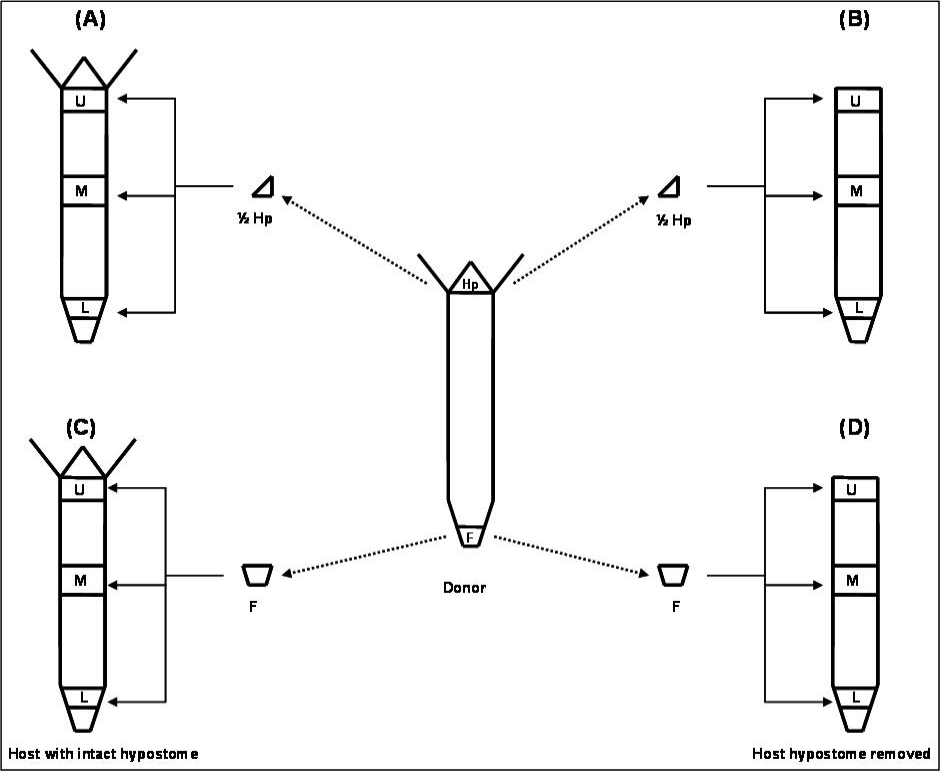
Lateral grafting procedure. A piece of hypostome (1/2 Hp) and a complete foot (F) used as transplants. The grafting was done in upper, middle and lower (U, M and L, respectively) parts of the host body column. To determine the role of host hypostome, it was either kept intact (A, C) or removed (B, D

Adult budding hydra were used for lateral grafting (1,2). Either a piece of hypostome or a complete foot was used as transplant (Fig.1). Grafting was done on three different regions of the host body column (U, L, M; Fig.1). ‘U’ represents upper part of the body column, the region just below the tentacular ring, ‘L’ represents the lower region, just above the foot while ‘M’ represents the middle part of the body column, the region just above the budding zone (Fig.1).

**Fig 2:**
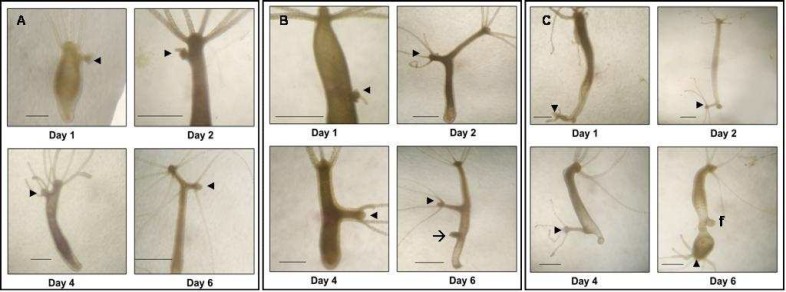
Grafting of hypostome. (A) A piece of hypostome grafted in upper region. (B) A hypostome piece grafted in middle part of the body column. (C) A piece of hypostome grafted in lower part of the body column. In all cases hypostome piece induces secondary axis (arrowheads). The arrow in B indicates an emerging bud. In C, f indicates foot of host. Scale bar =1mm

To determine the role of host hypostome on secondary axis, we used hosts either with or without intact hypostome (Fig.1). A piece of hypostome or foot was isolated from the donor by transverse cut made with a fine needle. An incision was made in the host ectoderm and transplant was inserted into the slit in such a way that a part of it protrudes out from the host. The host was left undisturbed for 2 to 3 hrs for the wound to heal. A small bulge in the region of the transplantation is an indication of graft acceptance. After 24 hrs, the first signs of induction were observed. The animals were routinely observed for secondary axis formation for at least 10 days. Except on the day of grafting, the grafted hydra were fed daily.

Two types of hosts, either with or without intact hypostome were used to examine the influence of host hypostome on the induction of the secondary axis. Grafting was carried out within and across species. In order to trace the migration of cells, either to or from the secondary axis, a combination of transgenic GFP-expressing H. vulgaris AEP hosts and non-transgenic H. vulgaris AEP donors were used.

## Results

**Induction capacity of hypostome within the species (homoplastic transplantation)**

The first indication of induction of a secondary axis was a localized swelling of the body wall that became evident within 2 to 3 hrs of grafting. In all, the three species used, namely, H. vulgaris Ind-Pune, H. magnipapillata and H. vulgaris, homoplastic transplantation resulted in induction of a secondary axis in over 90% of the cases (Table 1). About 5% of grafts apparently got assimilated which may be due to accidental insertion of transplants into the digestive tract of the host.

**Table 1:** Intraspecific hypostome grafts with host hypostome was left intact

**Hypostome Donor hydra**	**Host hydra with intact hypostome**	**Hypostome grafted on**	**No. of grafts done**	**No. of Positive grafts. Figures in the bracket indicates % graft accepted**
Hydra sp. India	Hydra sp. India	Upper	25	24 (96.00)
Hydra sp. India	Hydra sp. India	Middle	25	24 (96.00)
Hydra sp. India	Hydra sp. India	Lower	26	24 (92.30)
H. magnipapillata	H. magnipapillata	Upper	24	22 (91.66)
H. magnipapillata	H. magnipapillata	Middle	27	25 (92.60)
H. magnipapillata	H. magnipapillata	Lower	26	25 (96.15)
H. vulgaris	H. vulgaris	Upper	25	24 (96.00)
H. vulgaris	H. vulgaris	Middle	26	24 (92.30)
*H. vulgaris*	*H. vulgaris*	Lower	25	25 (100.00)

A piece of hypostome, when grafted in the upper region, induced a very small secondary axis with a short body column and hypostome with variable number of tentacles at the distal end (Fig.2A). When hypostome piece was grafted in middle part of the body column, relatively larger secondary axis was observed (Fig.2B). On grafting of the piece of hypostome onto the lower body part, a complete secondary axis with foot, body column, hypostome and tentacles was formed (Fig.2C). Thus, the host hypostome appears to exert an inhibitory effect on the induction of the secondary axis. However, not even a single case did we observe complete inhibition of induction due to host hypostome. After few days, the secondary axis stopped growing and started feeding independently. None of the grafts detached from the hosts up to ten

 days. The length of the secondary axis depends on the position of the graft, in that very small, moderate and complete axis was found to be formed in upper, middle and lower part of the body column, respectively. This indicates the presence of a head inhibitor (HI) as reported earlier (10,11). To confirm such an influence of the host hypostome, homplastic transplantations were carried out in hosts with their hypostomes completely removed. It was observed that secondary axis was induced in over 90% of the cases (Table 2).

**Table 2:** Intraspecific grafts performed after removal of host hypostome

**Hypostome Donor hydra**	**Host hydra without hypostome**	**Hypostome grafted on**	**No. of grafts done**	**No. of positive grafts. Figures in the bracket indicates % graft accepted**
Hydra sp. India	Hydra sp. India	Upper	25	24 (96.00)
Hydra sp. India	Hydra sp. India	Middle	26	26 (100.00)
Hydra sp. India	Hydra sp. India	Lower	22	20 (90.90)
H. magnipapillata	H. magnipapillata	Upper	24	23 (95.83)
H. magnipapillata	H. magnipapillata	Middle	22	20 (90.90)
H. magnipapillata	H. magnipapillata	Lower	25	24 (96.00)
H. vulgaris	H. vulgaris	Upper	26	25 (96.15)
H. vulgaris	H. vulgaris	Middle	24	23 (95.83)
H. vulgaris	H. vulgaris	Lower	24	22 (91.66)

**Table 3:** Hypostome grafted across the species with host hyposome left intact

**Hypostome Donor hydra**	**Host hydra with intact hypostome**	**Hypostome grafted on**	**No. of grafts done**	**No. of Positive grafts. Figures in the bracket indicates % graft accepted**
Hydra sp. India	H. magnipapillata	Upper	25	23 (92.00)
Hydra sp. India	H. magnipapillata	Middle	24	23 (95.83)
Hydra sp. India	H. magnipapillata	Lower	25	23 (92.00)
H. agnipapillata	Hydra sp. India	Upper	26	24 (92.31)
H. agnipapillata	Hydra sp. India	Middle	23	21 (91.30)
H. agnipapillata	Hydra sp. India	Lower	22	21 (95.45)

**Table 4:** Hypostome grafted across the species after removal of host hyposome

**Hypostome Donor hydra**	**Host hydra without hypostome**	**Hypostome grafted on**	**No. of grafts done**	**No. of positive grafts. Figures in the bracket indicates % graft accepted**	
Hydra sp. India	H. magnipapillata	Upper	25		23 (92.00)
Hydra sp. India	H. magnipapillata	Middle	24		22 (91.66)
Hydra sp. India	H. magnipapillata	Lower	25		24 (96.00)
H. magnipapillata	Hydra sp. India	Upper	25		23 (92.00)
H. magnipapillata	Hydra sp. India	Middle	25		25 (100.00)
H. magnipapillata	Hydra sp. India	Lower	25		24 (96.00)

**Induction capacity of hypostome piece across the species**

Interspecific (heteroplastic) transplantations were performed by using Hydra sp. India, and H. magnipapillata. Each of the species of hydra provided either the host or the transplant tissue. The slimy secretion that oozes out on piercing of endoderm of the host during incision seems to repel the transplant while grafting. However, this did not affect induction of secondary axis. In heteroplastic transplantations too, the incidence of secondary axis induction was very high (>90%; Table 3). Heteroplastic grafting performed after removing host hypostome also resulted in comparable inductions (Table 4).

**Induction capacity of foot within the species**

**Fig 3:**
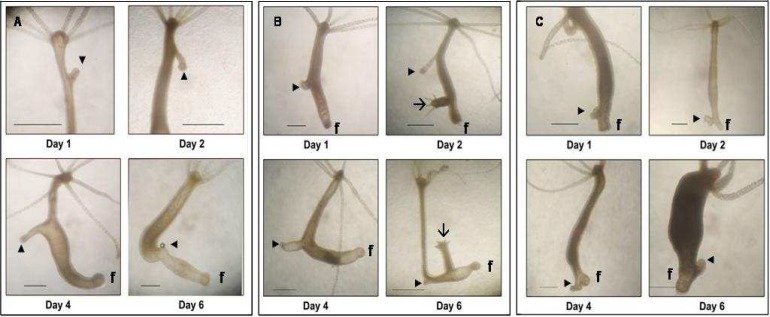
Grafting of a foot. A complete foot was isolated from host and grafted in upper (U), middle (M) and lower (L) part of the body column (A-C). Induction of small secondary axes with foot at their distal ends is indicated by arrowheads. Host foot is indicated by ‘f’. Arrow indicates a bud. Scale bar = 1 mm

**Fig 4:**
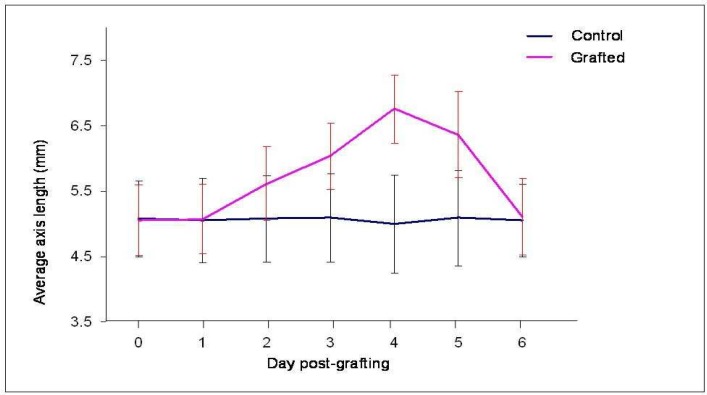
The effect of grafted foot on average length of the host. A complete foot was grafted in upper region of the host body column. The host hydra shows transient increase in the average length up to day four post-grafting. It attains normal length by day six post-grafting. Day 0 indicates the day of grafting

Foot and peduncle (region above the foot) have been shown to possess Organizing capacity for foot formation (3,6). We performed transplantations using foot as a transplant. The effect of host hypostome on secondary axis was checked as before by using two types of hosts, with hypostome or without hypostome. In intraspecific grafting, when a foot was grafted in upper part of the body column, it caused an induction. This small secondary axis has short body column (1-2 mm) with a sticky foot at the distal end (Fig.3A). Interestingly, the distance between host hypostome and induction caused by foot was found to increase till about day 4 post-grafting followed by a reduction that resulted in attaining the normal length by day 6 post-grafting (Fig.4). This phenomenon has appeared to have resulted from active repulsion between the two organizing centers residing in the hypostome of the host and the grafted foot. When foot was grafted in the middle part of the body column, induction having small body column and foot at the distal end has been observed (Fig.3B). Again the distance between host hypostome and new axis was found to increase on subsequent days. A foot was also found to induce an axis when transplanted in the lower part of the body column (Fig.3C). This new axis had very little or no body column with foot at the distal end; these axes often got fused with the host foot. All the inductions caused by a foot transplant had a sticky, apparently functional foot at the tip. When the induction capacity of foot within and across the species was compared, no difference was noticed as inductions were over 90% in both the cases. Removal of host hypostome did not influence the induction of secondary axis (Tables 5,6).

**Induction capacity of foot across the species**

Foot and peduncle of hydra have induction capacity within the species in that they induce proximal secondary axis (foot at the end of new axis) (6). By transplanting the foot of H. magnipapillata to Hydra sp. India and viceversa, we assayed its induction capacity.When the induction capacity of homospecific and heterospecific grafts was compared, no significant difference was found (Tables 7,8) the nature of the secondary axis was same in both cases.

**Table 5:** Foot grafted within the species in upper, middle or lower region of the host with host hypostome left intact

**Foot Donor hydra**	**Host hydra with hypostome**	**Foot grafted on**	**No. of grafts done**	**No. of positive grafts. Figures in the bracket indicates % graft accepted**	
Hydra sp. India	Hydra sp. India	Upper	20		18 (90.00)
Hydra sp. India	Hydra sp. India	Middle	20		19 (95.00)
Hydra sp. India	Hydra sp. India	Lower	21		20 (95.23)
H.magnipapilata	H.magnipapilata	Upper	22		20 (90.90)
H.magnipapilata	H.magnipapilata	Middle	20		19 (95.00)
H.magnipapilata	H.magnipapilata	Lower	22		21 (95.45)
H. vulgaris	H. vulgaris	Upper	20		19 (95.00)
H. vulgaris	H. vulgaris	Middle	21		20 (95.23)
H. vulgaris	H. vulgaris	Lower	20		19 (95.00)

**Table 6:** Foot grafted within the species in upper, middle or lower region of the host body column after removal of host hypostome

**Foot Donor hydra**	**Host hydra without hypostome**	**Foot grafted on**	**No. of grafts done**	**No. of positive grafts. Figures in the bracket indicates % graft accepted**
Hydra sp. India	Hydra sp. India	Upper	20	18 (90.00)
Hydra sp. India	Hydra sp. India	Middle	20	19 (95.00)
Hydra sp. India	Hydra sp. India	Lower	21	20 (95.23)
H.magnipapilata	H.magnipapilata	Upper	22	20 (90.90)
H.magnipapilata	H.magnipapilata	Middle	20	19 (95.00)
H.magnipapilata	H.magnipapilata	Lower	22	21 (95.45)
H. vulgaris	H. vulgaris	Upper	20	19 (95.00)
H. vulgaris	H. vulgaris	Middle	21	20 (95.23)
H. vulgaris	H. vulgaris	Lower	20	19 (95.00)

**Table 7:** Grafting of foot across the species in various regions of the body column with host hypostome

**Foot Donor hydra**	**Host hydra with intact hypostome**	**Foot grafted on**	**No. of grafts done**	**No. of positive grafts. Figures in the bracket indicates % graft accepted**
Hydra sp. India	H. magnipapillata	Upper	22	20 (90.90)
Hydra sp. India	H. magnipapillata	Middle	20	19 (95.00)
Hydra sp. India	H. magnipapillata	Lower	20	20 (100.00)
H. magnipapillata	Hydra sp. India	Upper	21	20 (95.23)
H. magnipapillata	Hydra sp. India	Middle	22	21 (95.45)
H. magnipapillata	Hydra sp. India	Lower	22	20 (90.90)

**Table 8:** Foot grafted across the species after removal of host hypostome

**Foot Donor hydra**	**Host hydra without hypostome**	**Foot grafted on**	**No. of grafts done**	**No. of positive grafts. Figures in the bracket indicates % graft accepted**
Hydra sp. India	H. magnipapillata	Upper	20	19 (95.00)
Hydra sp. India	H. magnipapillata	Middle	21	20 (95.23)
Hydra sp. India	H. magnipapillata	Lower	22	20 (90.90)
H.magnipapillata	Hydra sp. India	Upper	20	18 (90.00)
H.magnipapillata	Hydra sp. India	Middle	20	19 (95.00)
H.magnipapillata	Hydra sp. India	Lower	20	18 (90.00)

**Host supports the growth of secondary axis**

**Fig 5:**
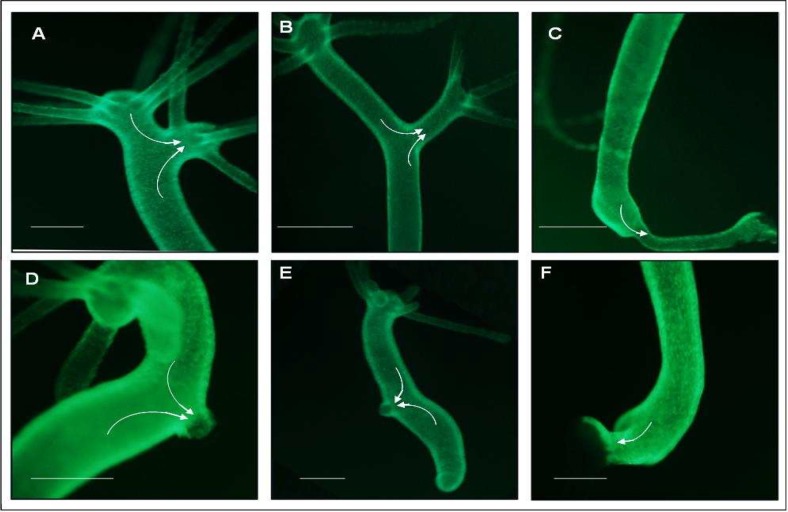
Contribution of host cells to the secondary axis. *H. vulgaris *(AEP) expressing GFP either in its ectodermal or endodermal cells were used as hosts and non GFP expressing transplants (a piece of hypostome or a complete foot) were grafted to induce secondary axis. A piece of hypostome (A-C) and complete foot (D-F) were grafted in the upper, middle and lower parts of the host body column. In all cases, ectodermal and endoderm supports the growth of the secondary axis

Hypostome has the characteristics of an organizer to induce host tissue to form most of the second axis. In contrast, tissue of the body column has a self-organizing capacity (it divides itself to form an axis) (7). By using stained transplant (hypostome and foot) and non-stained host, it is known that the signal(s) from these organizers can induce the host cells to participate in secondary axis formation (1, 2,7). We used H. vulgaris AEP strains (expressing GFP either in its ectodermal cells or in its endodermal cells) as a host and non-transgenic AEP as donor for our experiments. The transplantation of a foot and hypostome piece was carried out in different regions of the host body column. It was found that the cells (ectodermal and endodermal) from the host moved into the secondary axis and thus supported the growth of secondary axis (Fig.5). The transplant cells get replaced slowly by host cells as secondary axis grows.

**Multiple inductions**

**Fig 6:**
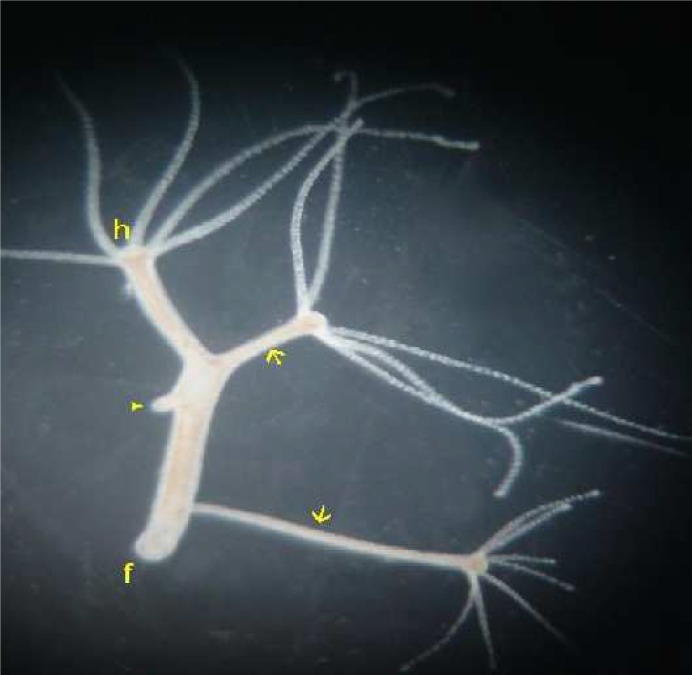
Multiple inductions by two pieces of hypostome and a foot in a single host. A foot was grafted in upper region of the body column, whereas two pieces of hypostome were grafted in middle and lower parts of the body column. Three secondary axes were induced. Relatively small secondary axis was induced by the foot (arrowhead). The distance between host hypostome (h) and grafted foot (arrowhead) went on increasing. Grafted foot is therefore seen in the middle part of the host body column. Two secondary axes were induced by pieces of hypostome in middle and lower parts of the host body column (two arrows). f indicates foot of the host. scale bar = 1 mm

In order to test whether multiple axes can be induced or not, we performed three intraspecific (H. magnipapillata) grafts on a single host. A complete foot and small pieces of hypostome were grafted in upper, middle and lower parts of the host body column, respectively. All three grafting procedures were completed in ten minutes. We obtained inductions in all the three grafted regions of the host. The length of the induction caused by a piece of hypostome is greater (2-4 mm) than that caused by the foot (up to 1 mm, Fig.6).

The length of hypostome-induced axes differed based on the position of the graft. As observed previously, the increase in total host length as well as distance between host hypostome and grafted foot was also evident here.

## Discussion

The hypostome and foot are the two extremities of the body axis of Hydra with a capacity to induce new axis on transplantation onto a host hydra. These two organizers have different properties. A small piece of hypostome can cause an induction (7) whereas, complete foot is required for the same. The length of secondary axis induced by the hypostomal piece is greater than that induced by foot at the same position. The distance between host hypostome and new axis caused by foot was found to increase on subsequent days. This migration stops once the new axis reaches the budding zone or below it. This may be due to the interaction between the two organizers that probably helps in maintaining a stable distance between the two extremities of the body. This may contribute significantly to the maintenance of body length in a given species of hydra.

Transplantation of a hypostome and foot within species invariably leads to the formation of a second axis (1,2,6). To demonstrate their induction capacity across the species, we used two species, H. magnipapillata and. Hydra vulgaris Ind-Pune. We found that both hypostome and foot can induce secondary axis across the species with same rate as within the species. The possible explanation for this could be that both Hydra vulgaris Ind-Pune and H. magnipapillata belonged to vulgaris group and Eurasian clade. Due to phylogenetical closeness, their organizer molecules may be conserved (8).

The length of secondary axis depends on the position of graft (1,2). HI from hypostome inhibits the formation of another hypostome and its concentration is graded down the body column (11), hence, the length of secondary axis may be taken as an indicator of positional cues like HI. This model of secondary axis may be useful in the study of pattern formation. Finally, by using transgenic hydra lines that express GFP, we have demonstrated that ectodermal and endodermal cells from the host hydra contribute to the formation of the induced axis. 

The present study thus demonstrates that a number of questions regarding cellular and molecular regulation of pattern formation can be addressed by using the time-tested technique of tissue grafting in hydra in combination with modern techniques in biology.
